# Evaluating the Role of Hydrophobic and Cationic Appendages on the Laundry Performance of Modified Hydroxyethyl Celluloses

**DOI:** 10.1021/acs.iecr.2c01698

**Published:** 2022-09-14

**Authors:** Marcellino D’Avino, Ruth Chilton, Si Gang, Mark R. Sivik, David A. Fulton

**Affiliations:** † Chemistry-School of Natural and Environmental Sciences, Newcastle University, Newcastle upon Tyne NE1 8QB, U.K.; ‡ Newcastle Innovation Centre, The Procter & Gamble Company, Newcastle upon Tyne NE12 9TS, U.K.; § Fabric & Home Care Innovation Centre, The Procter & Gamble Company, Cincinnati, Ohio 45202, United States

## Abstract

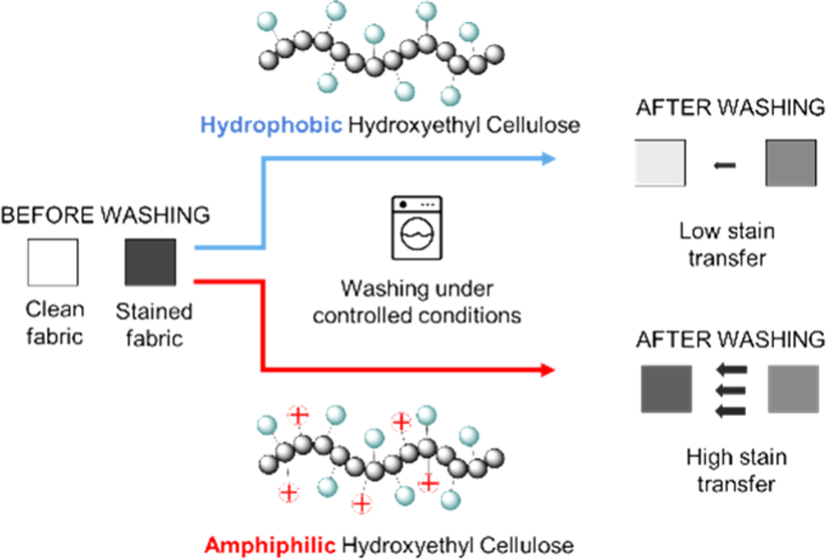

Soil-release polymers (SRPs) are essential additives of laundry detergents whose function is to enable soil release from fabric and to prevent soil redeposition during the washing cycle. The currently used SRPs are petrochemical-based; however, SRPs based on biorenewable polymers would be preferred from an environmental and regulatory perspective. To explore this possibility, we have synthesized SRPs based on hydroxyethyl cellulose (amphiphilic HEC) appended with controlled compositions of hydrophobic and cationic appendages and assessed their cleaning abilities. The results demonstrate that the introduction of hydrophobic lauryl appendages onto the HEC backbone is essential to deliver anti-redeposition and soil-release performance. Conversely, further introduction of cationic groups onto hydrophobic modified HECs had no clear impact on soil-release performance but caused significant disadvantages on anti-redeposition performance. We speculate that this poor performance arises on account of coacervation formation between the cationic HEC polymer and the anionic surfactant in the detergent, negatively impacting soil suspension and suggests that the inclusion of cationic appendages on HECs can ultimately lead to detrimental effects on performance. Interestingly, in contrast to conventional SPRs that exhibit good soil-release performance exclusively on synthetic fabrics, amphiphilic HEC displayed encouraging results on both synthetic and cotton-based textiles, possibly as a result of a good chemical affinity with natural fabrics. This work highlights that the nature and hydrophobic content of HEC ethers are key variables that govern HEC applicability as SRPs, thus paving the way for the design and synthesis of new SRPs.

## Introduction

The formulation of modern laundry products has dramatically changed through the last 20 years, mainly driven by changes in consumer washing habits, needs, and as a consequence of the evolution of regulations.^1^ Surfactants still represent the core of a laundry formulation; however, many other ingredients such as polymers, builders, bleaching agents, enzymes, and chelating agents have been introduced, thus enabling the fine control of cleaning performance.^2^ Water-soluble polymers play important roles in modern laundry products such as surfactant boosting, soil suspension, surface modification, dye transfer inhibition, etc. Initially, polycarboxylates, in the form of homo- and copolymers of acrylic acid and/or maleic acid, were introduced to replace sodium tripolyphosphate (which was generally used as a builder) to address government regulations and restrictions.^2^ Subsequently, a wide spectrum of other polymers was introduced into detergent formulations for different purposes (e.g., dye transfer inhibition, malodour control, fabric softening, etc.).

The use of soil-release polymers (SRPs) is a relatively recent advance that has followed the introduction of synthetic fibers into fabrics. Although the adoption of synthetic fibers (mostly polyester fibers) has led to more robust and resistant fabrics, it has also inevitably led to problems with oily stains. It is well-known that the wetting of oily stains (characterized by low surface energy) on low surface energy substrates (e.g., polyester textile) is thermodynamically favored. Once adsorbed on the surface, hydrophobic soil penetrates the fabric and is strongly retained by mechanical and electrostatic forces.^3^ Furthermore, since synthetic garments are highly hydrophobic, they are not wetted by the washing liquor, thereby making the removal of oily stains even tougher. Notwithstanding, soil adhesion and penetration can be controlled by modifying the surface energy or porosity of fabrics using either chemical or physical methods.^4^ Among chemical methods, plasma treatment is the most common, being a well-known procedure used to modify the surface structure of fabrics at the microscopic level (from angstrom to nanometers in thickness) without compromising the bulk properties.^5^ Plasma interacts with the textile surface by removing hydrogen atoms, thereby producing free radicals; these further react with oxygen resulting in the introduction of oxygen-containing functional groups onto the surface. As a result, fabric cleanability improves as a consequence of enhanced wettability and increased surface free energy, improving oil repellence.^5^ Among physical methods, fabric surface modification via SRPs has recently received great attention. Their mechanism of action is still to be understood, but it is presumed that it involves polymer adsorption on the fibers of fabrics.^1^ SRPs normally contain hydrophobic structural domains and hydrophilic structural domains. When synthetic fabrics are treated with a formulation comprising SRPs, the hydrophobic domains of SRPs are adsorbed onto the hydrophobic fibers, while the hydrophilic moieties stretch out toward the washing liquor. As a result of such absorption, the synthetic textile’s surface assumes a more hydrophilic finish. This offers two benefits: anti-redeposition and soil release. The former is important during a laundry washing process, where stains washed off from dirty garments tend to deposit back on fabrics. The formation of a hydrophilic film on synthetic textiles as a result of SRP deposition prevents stains from resettling on fabrics.

In the latter, deposition of SRPs inhibits soil from tightly adhering to fabrics. Indeed, soil sticks on top of the SRP film so that, during the next wash cycle, the adsorbed SRP layer desorbs from the fabric surface, thus removing also the stain layer. In addition to anti-redeposition and soil release, SRPs can also provide other benefits such as malodour prevention^6^ and in-wear comfort.

The most widely used SRPs in consumer detergent systems are polyesters based on terephthalate, namely, copolymer of poly(ethylene terephthalate) and poly(oxyethylene terephthalate) (PET-POET copolymer).^7^ These macromolecules possess a chemical architecture that comprises hydrophobic domains (the terephthalate structural units) that mimic the chemical structure of polyester fibers (PET fabrics) and hydrophilic domains made up of polyethylene oxide groups. The former are responsible for the adsorption of the polymer onto polyester fibers, while the latter promote the desorption of the adsorbed stain and polymer layer since they exhibit a higher affinity with the washing liquor.^8^ Like many other polymers used in laundry detergents, such as polycarboxylates, PET-POET copolymers are predominately petrochemical-based. Concerns regarding the irreversible depletion of fossil resources and evolving government policies as well as environmental concerns are driving the search for more sustainable alternatives. Polysaccharides are biorenewable or largely biodegradable, and consequentially, they have been a subject of a recent interest in the detergent industry. However, pure natural cellulose polymers do not possess the necessary physicochemical properties required for laundry applications and hence opportune chemical modifications are required to provide them with useful characteristics.

Among all polysaccharides, the chemical modification of cellulose (the most abundant polysaccharide on Earth) is by far the most extensively explored, more than any other natural polymer. Cellulose consists of linear chains of β (1 → 4)-linked d-glucose units, and due to its extended network of hydrogen bonds between and along each polymer chain, cellulose exhibits a crystalline structure that makes it practically insoluble in almost every organic solvent. Therefore, cellulose functionalization has been a key tool to enhance its solubility and to endow specific properties. As an example, cellulose hydroxyalkyl ethers such as hydroxyethyl (HEC) or hydroxypropyl (HPC) cellulose are highly soluble in water and are currently used as emulsifiers and thickeners.^9^ Cellulose derivatives, such as carboxymethyl cellulose (CMC), have been used in laundry formulations for over a decade.^10^

More recently, modified HECs appended with multiple hydrophobic and cationic appendages have been reported^11^ in the patent literature to be an effective SRP for both synthetic and natural fibers. Although its performance as a soil removal agent is described, there is still a lack of understanding about the way other aspects of the laundry washing process (e.g., anti-redeposition, interactions with other detergents ingredients, etc.) are affected in the presence of modified HECs with both hydrophobic and cationic appendages. This work aims to provide a better understanding of the capacity of modified polysaccharides to act as a laundry aid. Libraries of HEC derivatives featuring controlled compositions of hydrophobic and cationic appendages were synthesized and characterized. Their cleaning performance in terms of soil release and soil anti-redeposition was explored and the relationship between appendage compositions and washing performance was investigated. It was found that hydrophobic modified HEC possessing lauryl appendages exhibited good soil-release and anti-redeposition performance. By contrast, the addition of cationic groups onto hydrophobic HECs had no clear impact on soil-release performance but caused significant disadvantages on anti-redeposition, probably as a consequence of coacervation formation between cationic modified hydrophobic HECs and surfactants. Interestingly, unlike conventional SRPs that show soil-release benefits solely on synthetic fabrics, hydrophobic and cationic modified HECs herein exhibited clear soil-release performance even on cotton-based textiles.

**Figure 1:**
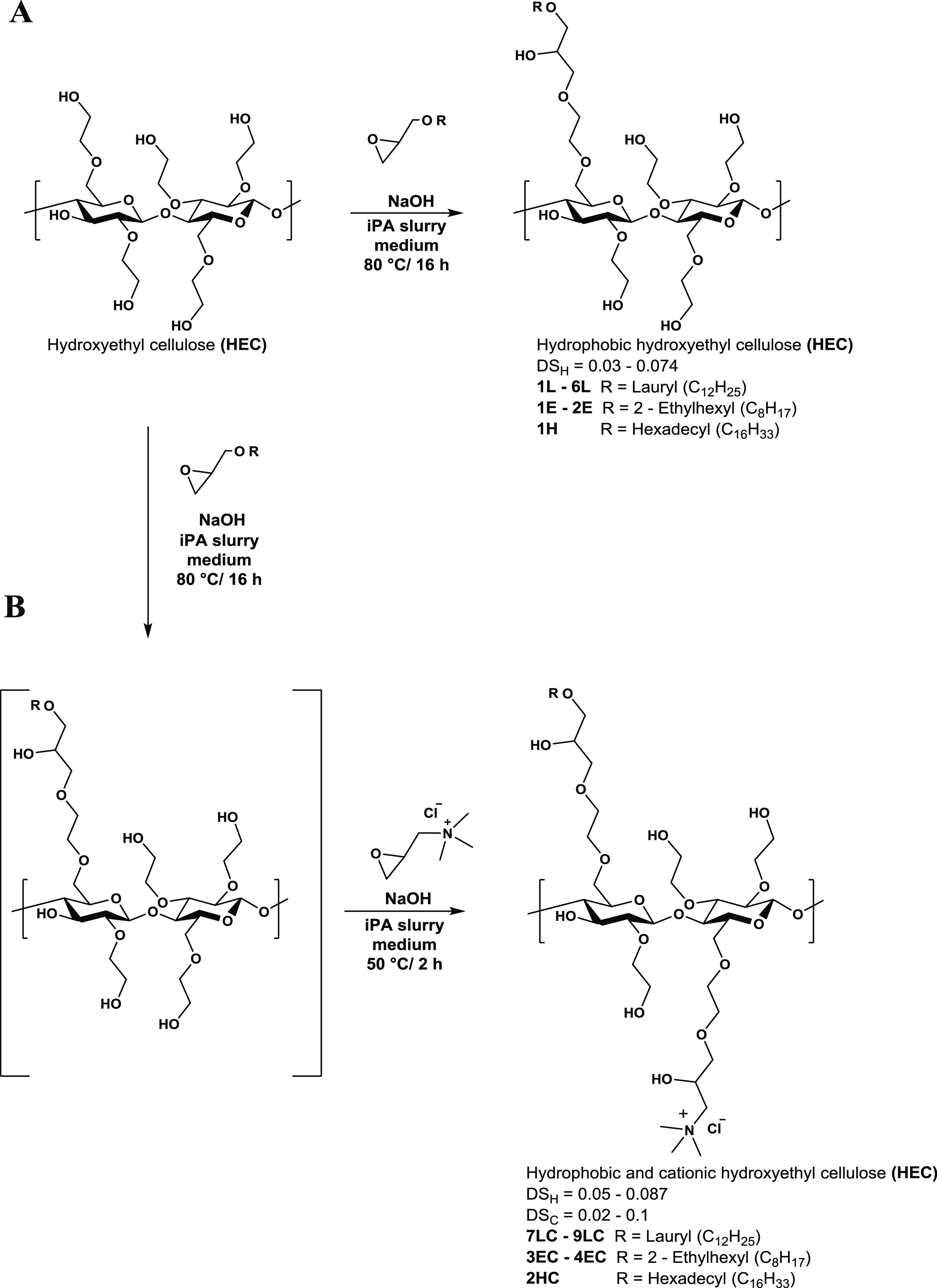
Synthesis of (A) hydrophobic modified HECs (**1L–6L**, **1E–2E**, **1H**) and (B) hydrophobic and cationic modified HECs (**7LC–9LC**, **3EC–4EC**, **2HC**).

## Experimental Section

### Materials and Methods

Hydroxyethyl cellulose, (HEC) commercially available as Natrosol 250 GR, was purchased from Ashland. According to the supplier information, this HEC is characterized by an average molar substitution of 2.5 (moles of ethylene oxide per single anhydroglucose unit) and an average molecular weight of approximately 300,000 Da. 2-Propanol (>99%), acetone (>99%), glacial acetic acid, sodium hydroxide, glycidyl trimethyl ammonium chloride (GTAC, >90%), hexadecyl glycidyl ether (GHE, >98%), and dialysis membrane tubing (3500 Da MWCO) were purchased from Sigma-Aldrich. Lauryl glycidyl ether (LGE, >98%) and 2-ethylhexyl glycidyl ether (GXE, >98%) were purchased from TCI chemicals. Dirty motor oil was acquired from Warwick Equest. A typical laundry formulation without soil-release polymers was provided by P&G (Newcastle Innovation Centre). All chemicals were used without further purification.

#### Synthesis of Hydrophobic HEC, **3L**

A 100 mL round-bottom flask equipped with a magnetic stirrer and condenser tube was charged with HEC (6 g, 21.85 mmol), 50 mL of an isopropyl alcohol/water solution (85:15), and an aqueous solution (0.720 g, 48% w/w) of sodium hydroxide. The slurry was stirred at room temperature for about 30 min under a nitrogen atmosphere. Then, lauryl glycidyl ether (0.26 g, 1 mmol) was added dropwise via a syringe and the reaction was heated at 80 °C for 16 h. After cooling to room temperature, the reaction mixture was neutralized with glacial acetic acid and the crude product was collected by vacuum filtration. The product was washed with an acetone/water solution (150 g, 80:20) and acetone (150 g) and then dried at 70 °C for 24 h under reduced pressure. Modified HEC was dissolved in 70 mL of water and mixed thoroughly at 65 °C overnight. The obtained gel-like solution was dialyzed against water for 2 days, and then freeze-dried to afford a pale-yellow solid (4.51 g, 70%). Samples **1L–6L**, **1E–2E**, and **1H** were obtained similarly by changing the type and amount of alkyl epoxide used (see Table 3).

#### Synthesis of Hydrophobic and Cationic HEC (**7LC**)

A 100 mL round-bottom flask equipped with a magnetic stirrer and condenser tube was charged with HEC (6 g, 21.85 mmol), 50 mL of an isopropyl alcohol/water solution (85:15), and an aqueous solution (0.720 g, 48% w/w) of sodium hydroxide. The slurry was stirred at room temperature for 30 min under a nitrogen atmosphere. Then, lauryl glycidyl ether (0.26 g, 1 mmol) was added dropwise via a syringe and the reaction was heated at 80 °C for 16 h. The temperature was reduced to 50 °C and glycidyl trimethyl ammonium chloride (0.96 g, 6.3 mmol) was added dropwise with a syringe and the mixture was allowed to react for 2 h to afford a yellow slurry. Modified HEC was dissolved in 70 mL of water and mixed thoroughly at 65 °C overnight. The obtained gel-like solution was dialyzed against water for 2 days, and then freeze-dried to afford a pale-yellow solid (4.50 g, 71%). Samples **7LC–9LC**, **3EC–4EC**, and **2HC** were obtained similarly by changing the type and amount of alkyl epoxide used (see Table 3).

### Characterization

^1^H NMR spectra were recorded on an Avance Bruker 700 MHz spectrometer operating at 25 °C. Samples were prepared by dissolving 5 mg of the product in 600 μL of D_2_O. Dissolution occurred immediately and clear solutions were obtained. The degree of substitution of the hydrophobic moieties (DS_H_) was measured by comparing the integration of the signal for the protons of the lauryl, hexadecyl or 2-ethylhexyl group of the alkyl chain to the H-1 anomeric signal of the glucose unit, which was integrated to 1 proton.

Fourier transform infrared (FTIR) spectra (in the range of 400–4000 cm^–1^ wavenumbers) were recorded using an IRAffinity-1S Fourier transform infrared spectrophotometer equipped with an attenuated total reflectance (ATR) sampler at 4 cm^–1^ spectral resolution. For each experiment, 50 scans were collected and averaged. Samples were dried overnight in a vacuum oven at 70 °C before each measurement.

Elemental composition was evaluated using a Thermo Fisher Scientific CHN elemental analyzer. From the measured N%, the cationic degree of substitution (DS_C_) was calculated according to eq 1 where *M*_RU_ is the molar weight of the repeating unit of HEC and *M*_GTAC_ is the molar weight of the cationic substituent^12^
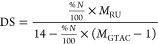
1

### Soil-Release Performance Test

Polyester (PE) and knit cotton (KC) fabrics were purchased from WFK Testgewebe GmbH. These were cut into 5 × 5 cm^2^ pieces and conditioned with modified HEC solutions in an automatic tergotometer as follows. Stock solutions of modified HEC were prepared in Milli-Q water (5% w/w). The latter were further diluted with hard water (21 gpg) into the tergotometer chambers to 50 ppm and mixed at 200 rpm for 10 min. Subsequently, fabrics were added and mixed thoroughly for 40 min at 35 °C followed by two 5 min rinse cycles using hard water (21 gpg). Fabrics were then dried overnight under humidity and temperature control (50% RH, 20 ± 2 °C). A list of the fabrics tested and the samples used is reported in Table 1.

**Table 1:** List of HEC Ethers Tested for Stain Removal with Each Type of Fabric Investigated

type of fabric	sample tested
knit cotton (CK)	**3L–6L**; **7L–9LC**
polyester (PE)	**3L**; **7LC**

**Figure 2:**
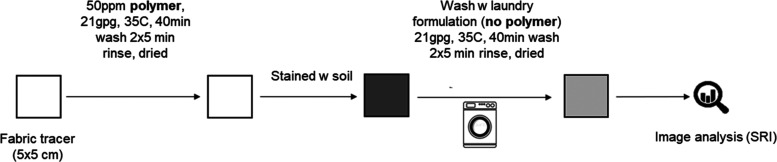
Summary scheme of the soil-release test conditions.

Soil-release performance tests were performed to assess the effect of textile surface modification by HEC ethers on the stain removal performance. Fabrics conditioned with modified HEC solutions were treated with dirty motor oil. Dirty motor oil (200 μL) was applied onto each square of fabric that were then dried overnight. Stain removal tests were executed in an automatic tergotometer. Stained garments were washed using a laundry detergent formulation that did not contain soil-release polymers for 40 min at 35 °C followed by two 5 min rinse cycles using hard water (21 gpg). Four replicates were run for each experiment. Stain removal performance was evaluated through image analysis. Stain images were collected before and after washing against a white background with a reflection spectrophotometer (DigiEye). Images were analyzed using DigiEye software. For each fabric, the color of the motor oil stains was evaluated by measuring the coordinates *L*_n_^*^, *a*_n_^*^, and *b*_n_^*^ defined in the CIELAB color system. From the measured coordinates, the differences in lightness (Δ*L*_n_^*^), redness (Δ*a*_n_^*^), and blueness (Δ*b*_n_^*^) in contrast to the background were calculated.^4,13^ The relative color changes, ΔE*, were calculated comparing the variation of the coordinates before (*n* = 1) and after the washing cycle (*n* = 2) by applying the following equation
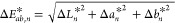
2Lastly, the stain removal index (SRI) was assessed as follows
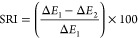
3A summary of the test conditions is displayed in Figure 2.

### Anti-Redeposition Performance Test

Polyester sheets loaded with BS2004 soil (SBL) were acquired from WFK Testgewebe GmbH and cut into 5 × 5 cm^2^ pieces. BS2004 is a synthetic soil mixture mainly composed of vegetable oil, synthetic sebum, and solid particles such as kaolin and carbon black. Polyester (PE), knit cotton (KC), polycotton (PC), and polyspandex (PS) fabrics were purchased from WFK Testgewebe GmbH and cut into 5 × 5 cm^2^ pieces. Whiteness tests were performed in an automatic tergotometer. Washing loads were composed of four specimens (tracers) for each type of fabric (PE, KC, PC, and PS): an adequate number of SBL swatches to simulate the soil levels of typical consumers and an appropriate amount of KC and PC garments in which the tracers were diluted to reproduce the washing conditions of the consumers. Stock solutions of modified HEC were prepared in Milli-Q water (5% w/w). The latter were further diluted in hard water (21 gpg) into the tergotometer chambers to 50 ppm together with a proper amount of a typical laundry detergent formulation and mixed at 300 rpm for 1 min. Subsequently, fabrics were added and mixed thoroughly for 40 min at 35 °C followed by two 5 min rinse cycles. Fabrics were collected from each chamber. Exhausted SBL swatches were replaced with fresh ones. A new washing cycle was performed under the same conditions reported above. The process was repeated four times in total. Lastly, tracers were separated from all other garments and dried overnight under humidity and temperature control (50% RH, 20 ± 2 °C).

**Figure 3:**
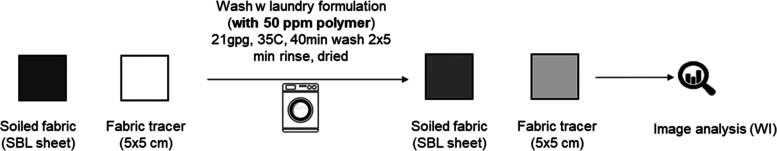
Summary scheme of the anti-redeposition test conditions.

The whiteness degree of a textile measures the capacity of a detergent to inhibit soil adsorption onto fabrics and to prevent soil redeposition during the washing cycle. The whiteness degree of fabrics was evaluated through image analysis. Tracers images were collected before and after washing with a reflection spectrophotometer (Konica Minolta: CM-3630A). Images were analyzed using SpectraMagicNX software. The color of each fabric was evaluated by measuring the coordinates *L*_n_^*^, *a*_n_^*^, and *b*_n_^*^ defined in the CIELAB color system. From the measured coordinates, the International Commission on Illumination (CIE) whiteness index (WI) was calculated by applying the following equation
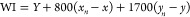
4where *Y* is the luminance factor while *x* and *y* are the color coordinates of the observed garment defined in the *Yxy* color space. These can be easily obtained by opportunely converting the measured values of *L*_n_^*^, *a*_n_^*^, and *b*_n_^*^, which correspond to *Y*, *x*, and *y*, respectively. *x**_n_* and *y_n_* are the color coordinates of the lighting source used. The WI values of tracers (fabric garments) washed solely with a laundry detergent formulation were used as negative control. More specifically, whiteness results were expressed as the difference (ΔWI) between the WI of tracers washed with modified HECs together with the detergent formulation and the WI of tracers washed with the detergent formulation only. High ΔWI values correspond to high soil anti-redeposition performance. A summary of the test conditions is displayed in Figure 3. Three whiteness tests were performed: test A, test B, and test C. In each test, different modified HEC batches were investigated. The list of the samples tested is shown in Table 2.

**Table 2:** List of HEC Ether Samples Tested for Whiteness

type of test	sample tested
test A	**3–6L**, **7–9LC**
test B	**1E–2E**, **3EC–4EC**, **1H**, **1HC**
test C	**1L–3L**

### Clay Suspension Stability

The ability of HEC ethers to maintain clay particles in suspension was investigated using a Turbiscan optical analyzer (Formulaction, L’Union, France). This instrument was equipped with a near infrared light source (880 nm) and two detectors working simultaneously. One detector measures the light flux transmitted (T) through the vial containing the sample, while the other monitors the backscattered light (Bs) at 135°. Samples **3L** and **7LC** were chosen as representative of hydrophobic and amphiphilic HECs, respectively. Stock solutions of modified HEC and of a typical laundry detergent formulation were prepared in Milli-Q water (0.6% w/w and 0.8%, respectively). A volume of 0.5 mL of these stock solutions was transferred to a 30 mL glass vial containing 0.06 g of clay. Hard water (9 gpg) was added to bring the total volume of the solution to 20 mL. The final concentrations of the modified HEC and clay were 150 and 3000 ppm, respectively, and the concentration of the laundry detergent was either 200 or 2000 ppm (in this case, the amount of stock solution used was 5 mL). Subsequently, the vial was sonicated in an ultrasonic bath for 15 min at 35 °C. Immediately after the sonication, the clay suspension was monitored by collecting T and B profiles every min for 1 h. The stability of each suspension was quantitatively evaluated by the Turbiscan Stability Index (TSI), a parameter used to study the destabilization phenomenon occurring in a colloidal system.^14^ The TSI is based on an integrated algorithm that takes into account the evolution of T or B signals over time and along the height of the vial. It is calculated according to the following equation
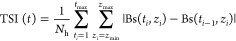
5where *z*_max_ and *z*_min_ are the higher and lower limits delimiting the area of the vial’s height where the TSI is calculated; *N*_h_ is the number of points along the vial height; *t*_max_ is the time at which the TSI is quantified. High TSI values are typical of unstable systems experiencing destabilization processes such as creaming, coalescence, or sedimentation. Conversely, low TSI values arise from highly stable systems.

### Coacervation Formation of Modified HEC with a Laundry Detergent

The behavior of solutions containing a modified HEC and a typical laundry detergent were monitored using a Brinkmann PC-950 colorimeter equipped with a 76.2 cm optic fiber and a 2 cm stainless steel probe. Samples **3L** and **7LC** were chosen as representative of hydrophobic and amphiphilic HECs, respectively. Stock solutions of modified HEC were prepared in Milli-Q water (5% w/w). The latter were further diluted in 500 mL of hard water (9 gpg) into a 1 L beaker to 10 ppm. A stock solution of a typical laundry formulation was prepared in Milli-Q water (10000 ppm). A volume of 0.1 mL of this stock solution was transferred into a 1 L beaker containing the HEC solution. The mixture was allowed to stabilize for a few seconds and the corresponding optical transmission value, recorded by the colorimetric probe, was collected. Further stepwise additions of the laundry detergent stock solution were performed until its final concentration in the beaker was 4000 ppm. Finally, the obtained transmission values were reported as a function of the laundry detergent level (ppm).

### Streaming Potential: Sample Preparation and Theoretical Background

Polyester (PE) fabrics were purchased from WFK Testgewebe GmbH. These were cut into 5 × 5 cm^2^ pieces and boiled in Milli-Q water for 30 min. Later, they were conditioned with modified HEC solutions and a laundry detergent solution in an automatic tergotometer as follows. Stock solutions of modified HEC and of a typical laundry detergent were prepared in Milli-Q water (5% w/w). The latter were further diluted with hard (9 gpg) water into the tergotometer chambers to 50 ppm (modified HECs) and 2000 ppm (laundry detergent) and mixed at 200 rpm for 10 min. Subsequently, fabrics were added and mixed thoroughly for 40 min at 35 °C followed by two 5 min rinse cycles. Fabrics were then dried overnight under humidity and temperature control (50% RH, 20 ± 2 °C). The ζ-potential of fabrics was evaluated with a SurPASS 3.0 (Anton Paar GmbH) equipped with a cylindrical cell. A concentration of 1 mM KCl solution was used as the streaming medium. A pressure variation between 600 and 200 mbar was produced for each zeta cycle. Each measurement was performed at the natural pH of the streaming medium that was found to be around 6.5. Three independent samples were analyzed and two zeta cycles were executed on each garment. The ζ-potential values were collected and averaged to obtain the main value.

The streaming potential is an electrokinetic effect that occurs at a solid–liquid interface as a consequence of the relative movement of one phase over the other. The measurement of this electrokinetic effect is used for the determination of the ζ-potential of porous materials such as fabrics or powders. The ζ-potential is based on the electrochemical double layer (EDL) theory that describes the ion distribution induced by a charged surface in a solution.^15^ According to this theory, the surface charge is neutralized by counterions located within two regions at increasing distance from the solid surface known as the Helmholtz (or stationary) layer and diffuse layer. The Helmholtz layer, contains immobile ions that are not considered in thermal motion. Conversely, the diffusion layer, is characterized by a dynamic atmosphere of mobile ions. Hydrophobic surfaces, such as polyester fabrics, despite not having ionizable species, tend to exhibit negative surface charge as a result of the absorption of hydroxyl ions. The presence of a surface charge gives rise to an electric potential that decays as the distance from the charged surface increases. The ζ-potential is the electric potential at the shear plane or slipping plane that indicates the layer that separates mobile ions with those that are strongly bonded to the charged surface.^3^

Streaming potential is physically measured by forcing an electrolyte solution to flow tangentially to a target surface inside a proper cell. Consequently, counterions are moved in the direction of the liquid flow. The presence of an electronic circuit with a high internal resistance results in a charge separation that causes a back current that partially compensates the current associated with the movement of the ions. The net charge variation gives rise to an electric current *I*_str_ and an electrical potential *U*_str_ or streaming potential that can be easily measured. For hard materials possessing a planar surface, the ζ-potential can be quantified by applying the Helmholtz–Smoluchowsky equation
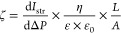
6where Δ*P* is the applied pressure, η is the viscosity of the streaming solution, ε is the dielectric coefficient of the electrolyte solution, ε_0_ is the vacuum permittivity, and *L*/*A* is the cell constant. The application of the Helmholtz–Smoluchowsky equation requires precise information concerning the geometry of the streaming channel (the space between two solid specimens), e.g., the cell constant.^16^

In the case of samples with irregular shapes and thus unknown cell constants, the streaming potential is usually quantified through the Helmholtz–Smoluchowsky approximation that does not require information about the geometry of the substrate according to the following equation
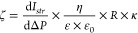
7where κ is the electrical conductivity.

## Results and Discussion

### Synthesis and Characterization

The synthesis of a series of functionalized HEC ethers (Figure 1) was prepared under heterogeneous conditions.^17,18^ Lauryl glycidyl ether (LGE), 2-ethylhexyl glycidyl ether (GHE), and hexadecyl glycidyl ether (GXE) were used as *O*-alkylating agents, while glycidyl trimethyl ammonium chloride (GTAC) was used as the cationizating agent. Hydrophobic appended HECs (hereinafter referred to as hydrophobic HECs, **1L–6L**, **1E–2E**, **1H**; Figure 1A) were obtained in a “one-pot process” by grafting hydrophobic groups onto the polysaccharide backbone through the reaction of HEC hydroxyl groups (whose nucleophilicity was activated by pretreating the HEC-isopropanol slurry with NaOH solution) with alkyl glycidyl ethers. Hydrophobic and cationic appended HECs (hereinafter amphiphilic HECs, **7LC–9LC**, **3EC–4EC**, **1HC**; Figure 1B) were obtained by first reacting HEC with alkyl glycidyl ethers and then subsequently adding GTAC to afford amphiphilic HEC. An idealized structure of modified HEC ethers is reported in Figure 1. It should be underlined that the modifying agents are reacted randomly amongst the available hydroxyl groups on the HEC’s backbone.^17^ Modified HECs displaying a wide range of DS were obtained by varying the amount of epoxide used. Details of the synthesized product are summarized in Table 3.

**Table 3:** Summary of Modified HECs Prepared by the Heterogeneous Etherification of Hydroxyethyl Cellulose Using Alkyl Glycidyl Ethers

	hydrophobic and amphiphilic hydroxyethyl cellulose						
product code^a^	DS_H_^b^	DS_C_^c^	yield (%)	HEC:GTAC^d^ (mol:mol)	HEC:alkyl^d^ (mol:mol) ether	product obtained (g)	*N*^e^ (%)
**1L**	0.071		57		1:0.120	3.22	
**2L**	0.026		66		1:0.080	3.56	
**3L**	0.017		70		1:0.040	4.51	
**4L**	0.007		61		1:0.020	3.87	
**5L**	0.005		64		1:0.015	3.94	
**6L**	0.003		65		1:0.010	4.02	
**7LC**	0.012	0.100	69	1:0.3	1:0.040	4.50	0.48
**8LC**	0.008	0.060	62	1:0.15	1:0.020	3.65	0.31
**9LC**	0.005	0.020	65	1:0.1	1:0.010	4.00	0.10
**1E**	0.044		46		1:0.100	2.95	
**2E**	0.033		50		1:0.050	3.38	
**3EC**	0.087	0.103	46	1:0.3	1:0.100	2.97	0.50
**4EC**	0.038	0.030	38	1:0.15	1:0.050	2.35	0.15
**1H**	0.026		58		1:0.050	3.64	
**2HC**	0.024	0.056	51	1:0.3	1:0.050	3.34	0.28

a **L**, lauryl glycidyl ether; **E**, 2-ethylhexyl glycidyl ether; **H**, hexadecyl glycidyl ether; **C**, glycidyl trimethyl ammonium chloride.

b Degree of substitution of hydrophobic moieties (DS_H_) calculated by NMR spectroscopy.

c Degree of substitution of cationic moieties (DS_C_) based on elemental analysis.

d Molar ratios between HEC and the modifying agents.

e Nitrogen percentage (N%) measured with elemental analysis.

**Figure 4:**
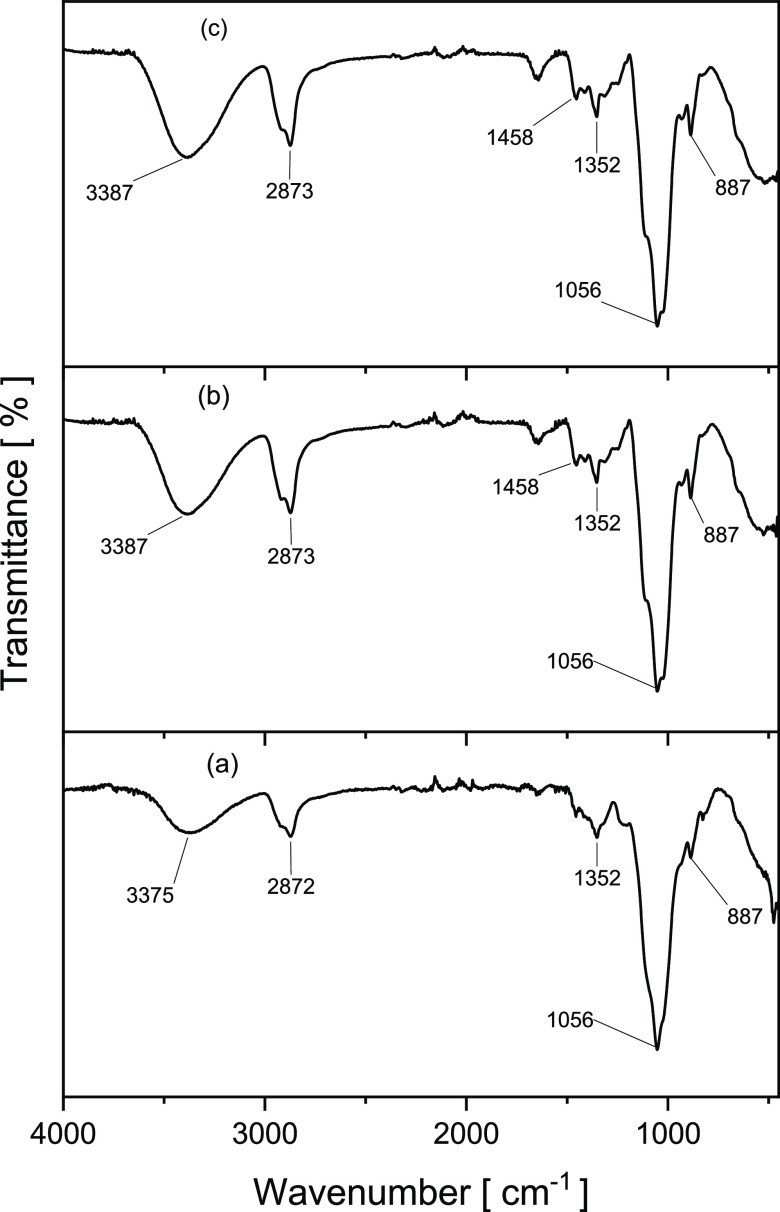
FTIR spectra of (a) hydroxyethyl cellulose (HEC), (b) hydrophobic modified HEC (**3L**), and (c) hydrophobic and cationic modified HEC (**7LC**).

The chemical structures of the modified HECs were studied by FTIR and ^1^H NMR spectroscopies. FTIR spectroscopy has proved to be a useful tool to evaluate polymer functionalization.^19^ As typical examples, FTIR spectra of pure HEC, hydrophobic HEC (**3L**), and hydrophobic and cationic HEC (**7LC**) are shown in Figure 4. Characteristic signals of the HEC scaffold were observed (Figure 4a–c) across all analyzed samples.^9,20,21^ The broad peak centered at ca. 3400 cm^–1^ corresponds to the stretching vibration of −OH; the band ranging 3000–2750 cm^–1^ was assigned to saturated C–H symmetric and asymmetric vibrations; the stretching vibration of C–O–C was observed at around 1050 cm^–1^ and the vibration of C–O glycosidic bond was observed at 887 cm^–1^. Successful etherification of the HEC backbone was confirmed (Figure 4b–c) by the appearance of a new band at 1458 cm^–1^ and the strengthening of the peak at 1352 cm^–1^, both of which correspond to the bending vibrations of methylene and methyl groups within the introduced saturated alkyl chains.^22^ No noticeable differences were observed (Figure 4b versus c) between FTIR spectra of **3L** and **7LC** (before and after the introduction of cationic moieties, respectively). Likewise, no remarkable differences in FTIR spectra were observed by changing the alkylating glycidyl ether used. This observation might be explained by the fact that all of the modifying agents possess similar chemical structures, and generally low level of modification (low DS_H_ and DS_C_).

**Figure 5:**
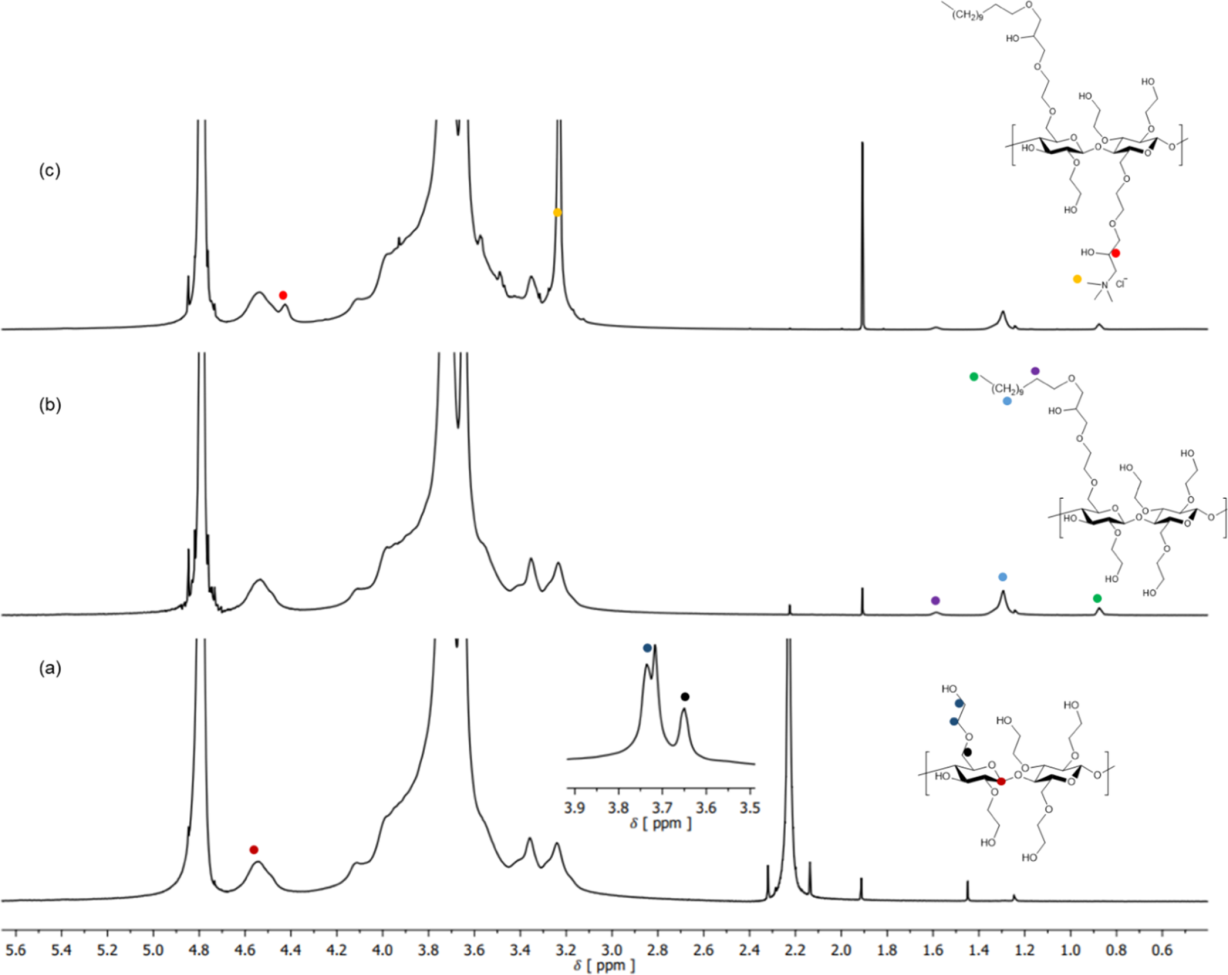
^1^H NMR spectra (700 MHz, D_2_O) of (a) hydroxyethyl cellulose (HEC), (b) hydrophobic HEC (**3L**), and (c) hydrophobic and cationic HEC (**7LC**).

The successful etherification of the HEC backbone was also confirmed by ^1^H NMR spectroscopy. The ^1^H NMR spectrum of unmodified HEC is shown in Figure 5a. The signal at 4.54 ppm corresponds to the anomeric proton (H-1) of the glucopyranose unit. The signals associated with the methylene protons of each hydroxyethyl unit and those related to the C-6 methylene protons were observed at 3.73, 3.71, and 3.65 ppm, respectively. The latter signals were observed to overlap with the unresolved broad proton signals of the glucopyranose units. Figure 5b depicts the ^1^H NMR spectrum of hydrophobic HEC modified with LGE (**3L**). A new series of signals were observed (1.5–0.8 ppm) corresponding to the alkyl protons of the lauryl chains alkylated onto the HEC backbone. The signal at 1.5 ppm was assigned to the methylene protons (labeled in purple) one carbon away from the oxygen atom of the glycidyl ether; the broad unresolved signal at 1.2 ppm (labeled in light blue) arises from the resonances of the (CH_2_)_9_ chain; the signal at 0.8 ppm represents the methyl protons at the terminus of the alkyl chain. Chemical modification of hydrophobic HEC with GTAC resulted in a sequence of new signals as observed in the spectrum of **7LC** (Figure 5c). Indeed, the small shoulder observed at 4.42 ppm corresponds to the methine proton of the glycidyl trimethyl ammonium chain, and the intense signal at 3.26 ppm was attributed to protons of the methyl groups of the quaternary ammonium. The DS_H_ of the hydrophobic moieties was calculated from each ^1^H NMR spectrum using the anomeric (H-1) proton as reference, whereas the DS_C_ of the cationic groups was evaluated from the percentage *N* measured by elemental analysis (see Table 3). Increasing the molar ratio between the modifying agents and HEC resulted in higher values of DS (Table 3) as highlighted by the strengthening of the intensities of the HEC side group signals in the NMR spectra (data not shown) and by the increasing percentage *N* measured by elemental analysis. NMR and FTIR spectroscopic analyses suggest that the synthesized HEC ethers are in agreement with the proposed structures and degrees of alkylation.

### Soil-Release Performance

**Figure 6:**
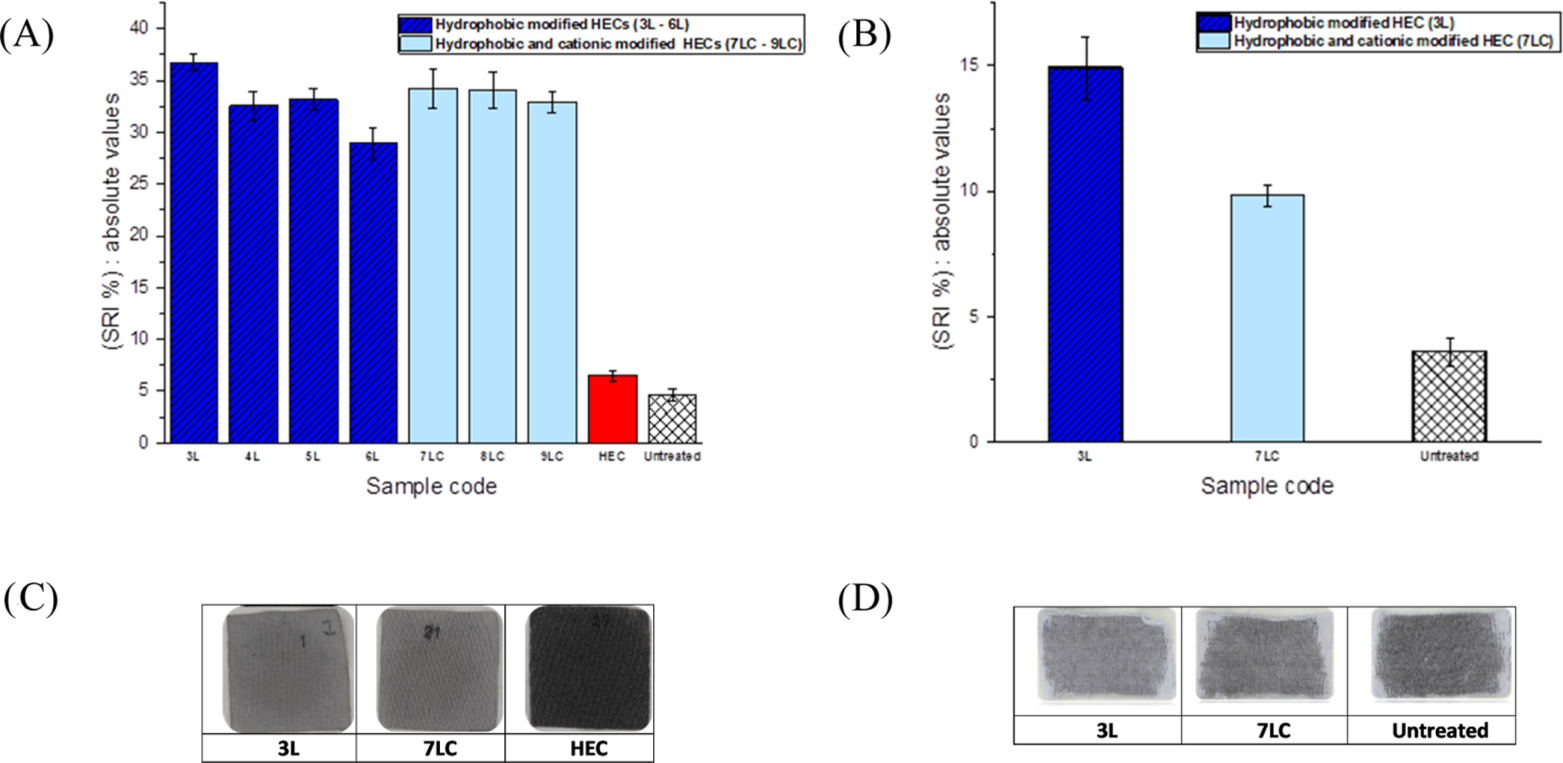
(A) Stain removal index (SRI) for polyester fabrics pretreated with hydrophobic modified HEC (**3L–9L**, blue), hydrophobic and cationic modified HEC (**7LC–9LC**, light blue), and unmodified HEC (red) compared with untreated fabrics (white). (B) Stain removal index for cotton fabrics treated with hydrophobic modified HEC (**3L**, blue) and hydrophobic and cationic modified HEC (**7LC**, light blue) compared with untreated fabrics (white). (C) Polyester fabrics were pretreated with pure unmodified HEC (left), hydrophobic HEC (**3L**, center), and amphiphilic HEC (**7LC**, right) and then stained and washed. (D) Cotton fabrics were treated with hydrophobic HEC (**3L**, center) and amphiphilic HEC (**7LC**, right) and then stained and washed.

Soil-release performance tests were performed on polyester fabrics with and without pretreatment with the HEC ethers that in these tests act as soil-release polymers (SRPs). Stain removal indexes (SRIs) were calculated through image analysis and are shown in Figure 6A. The SRI measured for polyester treated with unmodified HEC (red) was comparable with that recorded for the untreated polyester (white). This observation could be explained by the low affinity of pure HEC with synthetic fibers. Conversely, the introduction of hydrophobic modifications onto the HEC backbone (**3L–6L**) has a significant impact on soil-release performance as suggested by the high SRI values recorded (blue). Amphiphilic HEC carrying both hydrophobic and cationic modifications (**7LC–9LC**, light blue) appears to deliver similar levels of soil-release benefits as hydrophobic HEC does (light blue). This observation suggests that hydrophobic modifications play a substantial role in stain removal, while cationic moieties have little influence. Some images of tested polyester swatches after washing are depicted in Figure 6C where no remarkable difference arises between comparing polyester fabrics treated with **7LC** (amphiphilic HEC) or **3L** (hydrophobic HEC) as they look chromatically identical. Conversely, the appearance of the fabric washed with unmodified HEC is very different. Indeed, as indicated by the intense dark color of the textile, very little stain was removed from the textile surface, perhaps on account of weak adsorption of the corresponding SRP onto the textile surface.

The soil-release performance of modified HEC ethers on cotton fabrics was also evaluated. Samples **3L** and **7LC** were tested as representative hydrophobic and amphiphilic HECs, respectively. The stain removal test was performed under the same conditions as for polyester fabrics. Calculated SRIs are shown in Figure 6B. Overall, although SRI values for cotton textiles are lower than those of polyester, it is clear that modified HEC is able to provide soil-release benefits even on cotton. As shown in Figure 6B, the SRI values observed for hydrophobic HEC (**3L**) and amphiphilic HEC (**7LC**) are, respectively, two and three times higher than those recorded for unmodified HEC. Images of stained and pretreated cotton swatches after washing are shown in Figure 6D. It can clearly be observed that fabrics conditioned with modified HEC (especially **3L**) possess a less intense dark color as a result of their stain removal activity.

### Anti-Redeposition Performance

**Figure 7:**
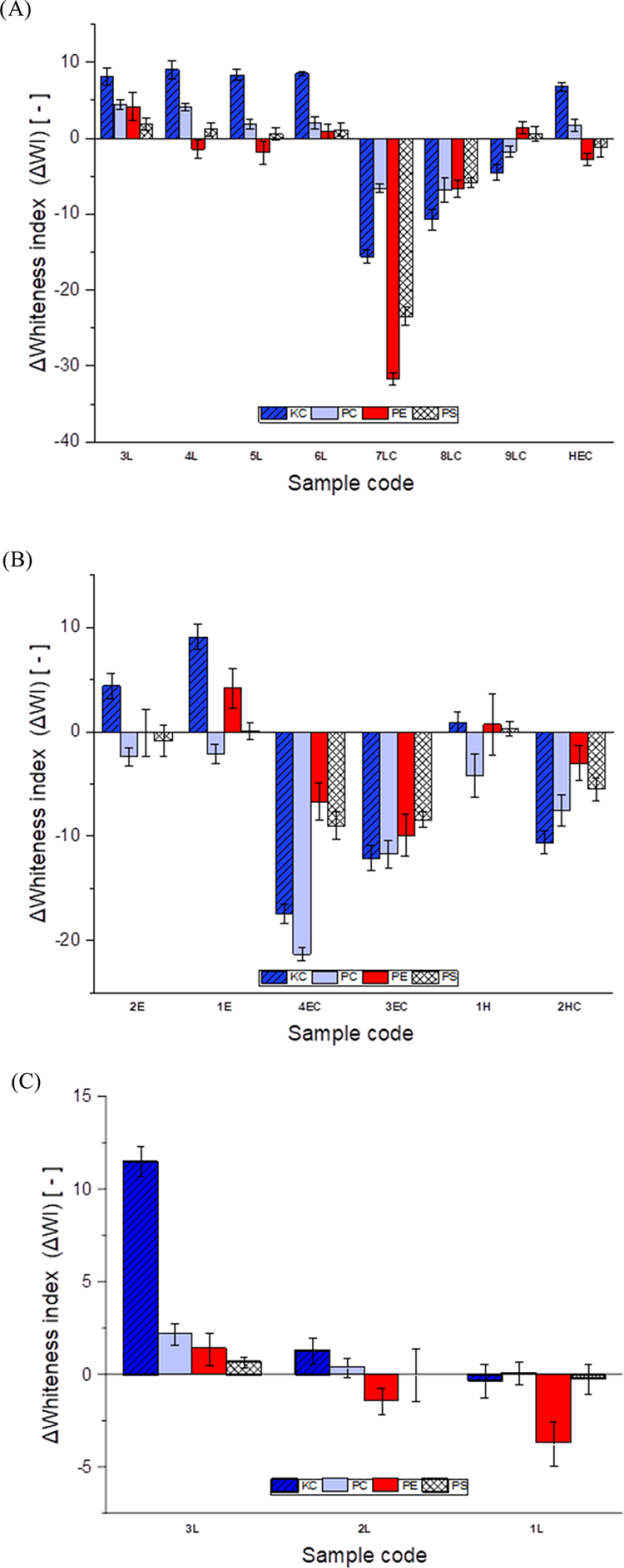
(A) Test A results: whiteness index variation (ΔWI) of knit cotton (blue, CK), polycotton (light blue, PC), polyester (red, PE), and polyspandex (white, PS) tracers washed with a laundry detergent formulation in the presence of HEC ethers (**3L–6L**, **7LC–9LC**). (B) Test B results: whiteness index variation (ΔWI) of knit cotton (blue, CK), polycotton (light blue, PC), polyester (red, PE), and polyspandex (white, PS) tracers washed with a laundry detergent formulation in the presence of HEC ethers (**1E–2E**, **3EC–4EC**, **1H**, **1HC**). (C) Test C results: whiteness index variation (ΔWI) of knit cotton (blue, CK), polycotton (light blue, PC), polyester (red, PE), and polyspandex (white, PS) tracers washed with a laundry detergent formulation in the presence of HEC ethers (**1L, 2L**, and **3L**).

In a typical anti-redeposition test, clean fabric tracers are washed together with stained fabrics in the presence of anti-redeposition additives. During the washing process, clean fabric tracers partially adsorb materials washed off from the stained fabrics. At the end of the washing cycle, the color variation of clean fabric tracers is quantified (ΔWI variation) to monitor the anti-redeposition performance of the additive. In this work, whiteness tests were performed on polyester (PE), knit cotton (KC), polycotton (PC), and polyspandex (PS) fabrics. The whiteness indexes (WIs) were calculated through image analysis. The results of test A are reported in Figure 7A. In test A, hydrophobic and amphiphilic HEC ethers possessing lauryl groups (**3L–6L**) only were tested. As shown in Figure 7A, unmodified HEC appears to deliver whiteness benefit on KC (labeled in blue) and PC (labeled in light blue) fabrics only. The introduction of hydrophobic lauryl modifications onto the HEC backbone (**3L–6L**) resulted in high ΔWI values especially for PC and KC. Together, these observations suggest that the addition of hydrophobic moieties seems not only to strengthen the benefit afforded by the HECs to cotton-based textiles but also provides a small benefit on synthetic fabrics such as PE and PS (labeled in red and white, respectively). Overall, sample **3L**, with the highest DS_H_ (0.017) is able to provide the best whiteness performance on all tested garments. Conversely, the addition of cationic modifications (**7LC–9LC**) has a significant negative impact on the anti-redeposition performance. Indeed, for all samples carrying both hydrophobic and cationic modifications, negative ΔWI values were registered. Interestingly, a consistent diminution of the whiteness index was observed for all types of fabrics when the content of cationic groups increased (from **9LC** to **7LC**). Negative ΔWI values result from a high adsorption of soil material onto the garment surface. We can speculate that this observation is a consequence of the weak capacity of amphiphilic HEC ethers to keep soil in suspension, thus leading to its precipitation in the washing liquor. On a molecular level, we can speculate that the addition of positively charged groups (via reaction with GTAC) onto the HEC backbone, exposes it to interact with other oppositely charged species within the washing liquor, i.e., anionic surfactants. Driven by electrostatic interactions, amphiphilic HEC and anionic surfactants form an insoluble colloidal inclusion or coacervate that compromises the capacity of the washing formulation to maintain the suspended soil. As a result, the latter precipitates and eventually deposits onto the fabric surface, causing the observed whiteness loss.

Test B was performed to further understand the effect of the nature of the hydrophobic moieties of HEC ethers on soil anti-redeposition. The effect of alternative hydrophobic groups was investigated by testing samples **1E–4EC** and **1H–2HC** carrying 2-ethylhexyl and hexadecyl modifications, respectively. Changing the hydrophobic moieties from lauryl to 2-ethylhexyl did not provide any noticeable advantage. Indeed, similar ΔWI values were recorded for samples **1E** and **2E**. No significant benefit was observed for sample **1H** that displays hexadecyl groups. Similar to that observed in test A, the introduction of cationic modifications (samples **3EC**, **4EC**, and **1HC**) causes significant whiteness loss.

Lastly, in test C, the effect of the DS_H_ on soil anti-redeposition was explored. To assess the impact of increasing the lauryl DS_H_ samples, **1L** (DS_H_ = 0.071), **2L** (DS_H_ = 0.026), and **3L** (DS_H_ = 0.017) were directly compared. As shown in Figure 7C, samples **1L** and **2L** were found to possess very low ΔWIs and therefore are less effective in soil anti-redeposition than sample **3L**. This observation, along with the results obtained in test A suggests that soil anti-redeposition benefits are only observed at an optimum DS_H_, namely, the lauryl content of modified HECs must be kept within a very specific and narrow range to deliver significant whiteness benefits.

### Clay Suspension Stability: Results

**Figure 8:**
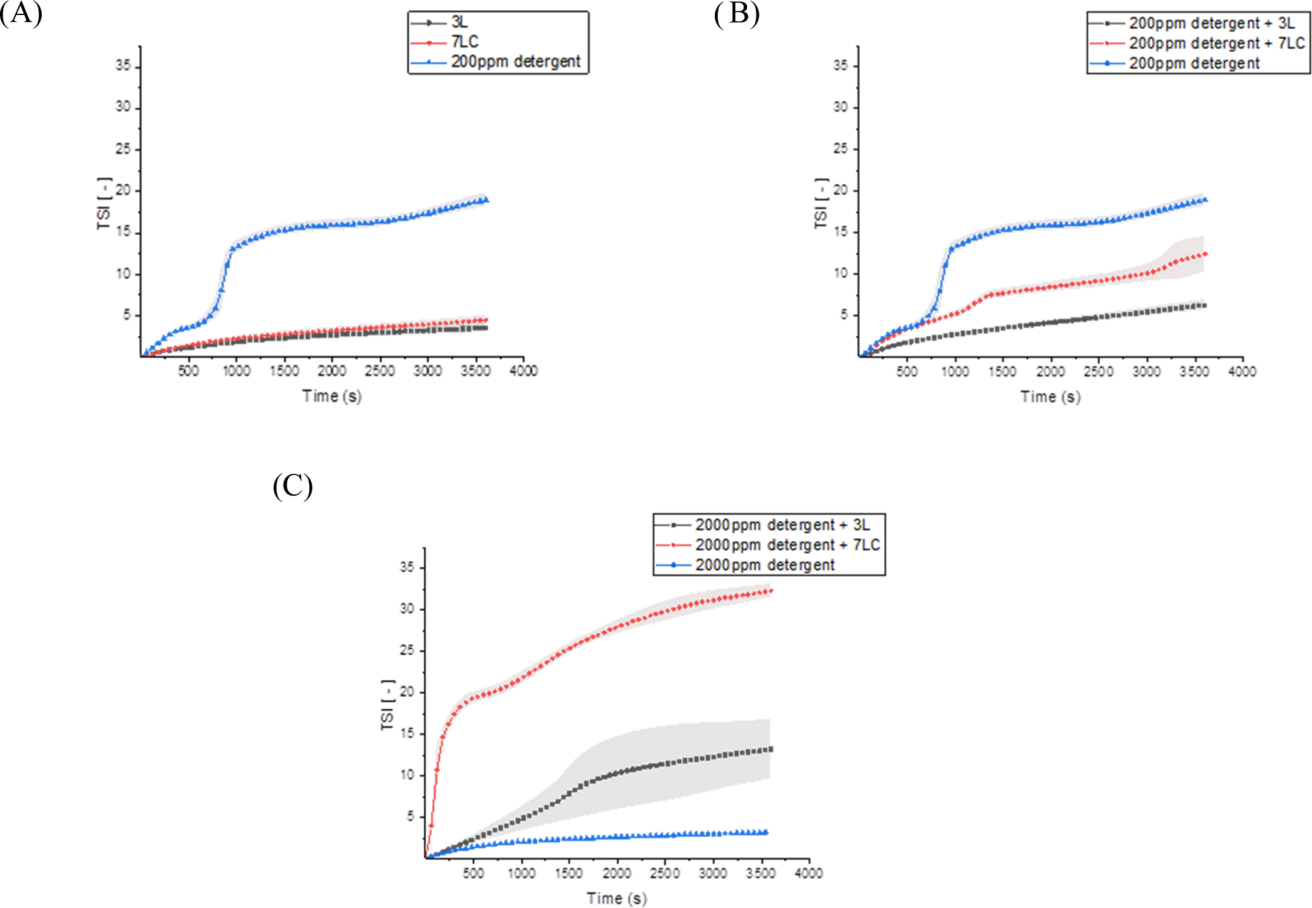
Turbiscan stability Index (TSI) as a function of time (s). (A) Evolution of the TSI index of samples **3L** and **7LC** compared with the TSI index of a laundry detergent (200 ppm) in the absence of polymers. (B) Evolution of the TSI index of a typical laundry detergent solution (200 ppm) with samples **3L** and **7LC** compared with the TSI index of a typical laundry detergent solution (200 ppm) in the absence of polymers. (C) Evolution of the TSI index of typical laundry detergent solution (2000 ppm) with samples **3L** and **7LC** compared with the TSI index of a typical laundry detergent solution (2000 ppm) in the absence of polymers.

The adsorption of particulate soil onto fabrics has been extensively studied over the past 50 years.^23^ The adsorption of hydrophilic solid particles, such as clay, is typically driven by multiple factors such as van der Waals forces, hydrogen bonds, or bridging by positively charged polyvalent ions. Although the interactions of clay particles with textile surfaces are decisive in conveying their adsorption, the stability of clay suspensions in the washing liquor is equally important. In an attempt to shed light on the observed anti-redeposition results, the ability of HEC ethers to maintain clay particles in suspension was explored. In this work, clay (in the form of kaolinite) is the main solid component of BS2004 soil used in whiteness tests. It is therefore crucial to understand clay suspension behavior in the presence of modified HEC solutions. Samples **3L** and **7LC** were chosen as representative of hydrophobic and amphiphilic HECs, respectively. Clay suspension stability was monitored by following the evolution of the Turbiscan Stability index (TSI) over 1 h. The results obtained are displayed in Figure 8.

In Figure 8A, the TSI of samples **3L** (150 ppm, red line), **7LC** (150 ppm, black line), and a typical laundry detergent (200 ppm, blue line) are shown. Samples **7LC** and **3L** revealed comparable trends with low TSI values observed with time. Conversely, the TSI of the laundry detergent greatly increased over the measurement time, thus indicating a highly unstable system. The TSI of clay suspensions of HEC ethers (150 ppm) in the presence of a laundry detergent (200 ppm) is depicted in Figure 8B and compared with the TSI of a laundry detergent only (200 ppm). In contrast with previous observations, the behaviors of samples **3L** and **7LC** were significantly different. Although the measured TSI values increased in both cases, that of the solution containing sample **7LC** witnessed a higher increment. This observation suggests that the addition of the laundry detergent resulted in less stable clay suspensions to a degree that depends on the type of HEC modification. Interestingly, as seen in Figure 8C, a further increase of the laundry detergent concentration (from 200 to 2000 ppm) caused a moderate destabilization for sample **3L**, and a complete phase separation was observed for sample **7LC** within the same time. Indeed, the TSI of amphiphilic HEC (red line) soared over the first 4 min and then steadily increased at a lower rate. By contrast, sample **3L** (black line) exhibited much lower TSI values, reflecting a more stable suspension. This observation confirms that the interactions of the detergent with hydrophobic or amphiphilic HECs play an important role in driving their ability to stabilize clay particle suspensions, especially for amphiphilic HEC that possess both cationic and hydrophobic appendages. Lastly, the laundry detergent alone at high concentration (blue line, 2000 ppm) displayed the lowest TSI values, thus suggesting that its capacity to maintain clay particles suspended is strongly influenced by the concentration.

### Coacervation Formation of Modified HEC with a Laundry Detergent: Results

**Figure 9:**
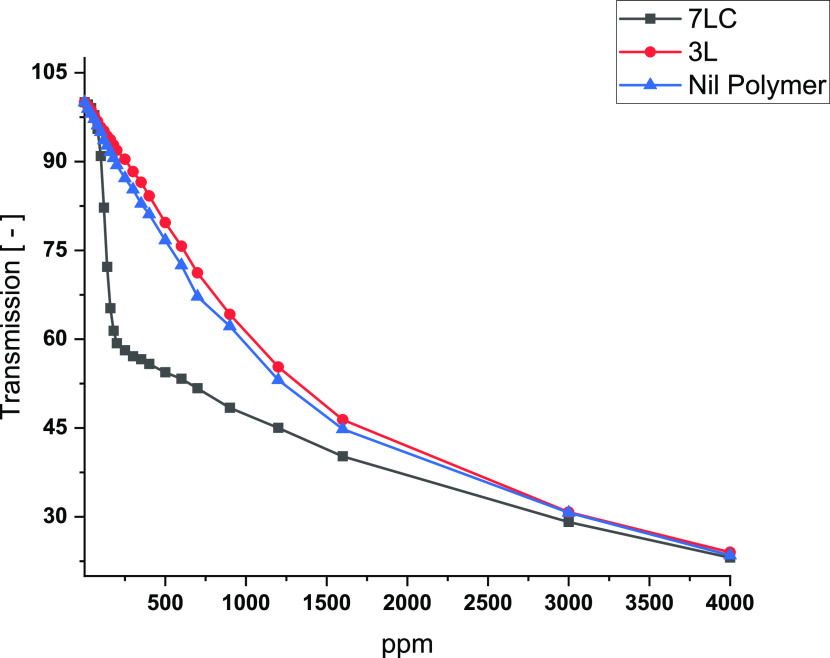
Transmission values for modified HEC solutions as a function of a laundry detergent concentration.

Laundry detergents typically contain mixtures of anionic and nonionic surfactants whose interactions with polyelectrolytes (e.g., cationic polymers) govern the stability and performance of the cleaning system under washing conditions. The behavior of aqueous solutions of oppositely charged species has been comprehensively studied.^24^ It is well-known that at a specific concentration (known as cac), surfactant aggregates start to bind to the polymer backbone, thus leading to a macroscopic associative phase separation (coacervate formation). Eventually, further increases in surfactant concentration result in the dissolution of the coacervate. The dissolution and precipitation process has been used as a tool to enhance deposition of insoluble cationic polymers and anionic surfactant complexes onto hard surfaces (e.g., fabrics).^25^ In this work, the behavior of solutions of modified HECs was explored by monitoring the changes in the transmitted light as a stock laundry detergent solution was titrated. The results are shown in Figure 9.

As shown in Figure 9, a consistent depletion of the transmitted light was observed in all experiments. In the absence of polymers (blue line), the transmitted light decreased as the concentration of surfactants increased, leading to a turbid solution (image not shown). This turbidity is typically due to the precipitation of insoluble calcium or magnesium salts of anionic surfactants in hard water.^26^ Sample **3L** (red line), which possesses hydrophobic groups only, exhibits the same behavior observed in the absence of polymers. Indeed, despite some small oscillations, the intensity of the transmitted light steadily falls. Conversely, the transmission observed for sample **7LC** (amphiphilic HEC, black line, Figure 9) displays a different trend. Initially, the transmission rapidly decreased, then, once the concentration of surfactants increased over 200 ppm, it continued to decrease but at a slower rate. This abrupt variation suggests that the behavior of this system is governed by two separate mechanisms that contribute to the observed transmission loss. First (when surfactant level < 200 ppm), amphiphilic HEC and anionic surfactants participate in the formation of an insoluble coacervate that causes a significant increase in the cloudiness of the solution. Subsequently (when surfactant level > 200 ppm), anionic surfactant salts precipitate, thus inducing a more moderate depletion of the transmission that occurs at a rate comparable with that registered for sample **3L**. This finding could explain the results observed in clay suspension tests, where it was found that amphiphilic HEC (sample **7LC**) in the presence of surfactants formed unstable clay suspensions under specific circumstances. More specifically, when the ratio between surfactants and amphiphilic HEC reached a certain value, insoluble complexes were formed between these two oppositely charged species, driving a macroscopic phase separation. Therefore, no active species were available to stabilize the suspension of clay particles suspended. As a consequence, clay particles rapidly aggregate and precipitate in the washing liquor.

### Streaming Potential: Results

**Figure 10:**
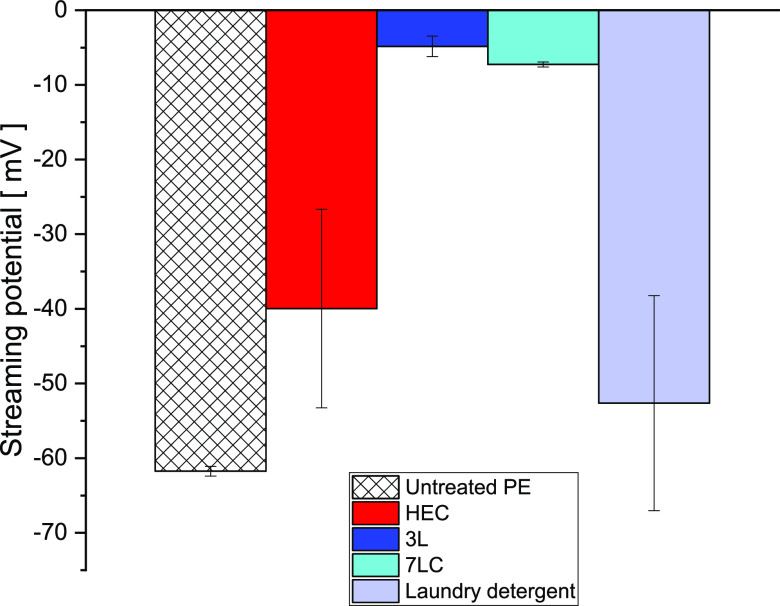
ζ-Potential values of polyester fabrics conditioned with modified HEC solutions. White: untreated polyester; red: unmodified HEC; blue: hydrophobic HEC (sample **3L**); cyan: amphiphilic HEC (sample **7LC**); light blue: laundry detergent.

A streaming potential study of polyester fabrics conditioned with modified HEC was performed. The streaming potential was measured with a Surpass 3.0 equipped with a cylindrical cell. In brief, a streaming medium is forced to flow through the cell containing a polyester tracer by applying a pressure gradient, causing the excess charges to move in the flow direction. The streaming potential is associated to the net charge separation occurring (in the measuring capillary) at equilibrium between the streaming current (excess charge circulation) and back current (caused by the presence of an electric circuit). Clean polyester fabrics were conditioned with unmodified HEC, samples **3L** or **7LC** (representatives of hydrophobic and amphiphilic HECs, respectively), and a typical laundry detergent. The results are shown in Figure 10.

The streaming potential for untreated polyester fabrics (white) was found to be ∼−60 mV, which is in agreement with previous findings.^3,4^ Although polyester textiles do not possess ionizable species, these tend to exhibit a negative potential as a result of the preferential adsorption of hydroxyl ions.^3,4^ Polyester fabrics conditioned with unmodified HEC displayed a ζ-potential of approximately −40 mV (red). The increase in the potential value arises on account of the adsorption of HEC chains onto the synthetic textiles causing a partial displacement of the adsorbed hydroxyl ions. A further increase of ζ-potential with respect to untreated fabrics was registered for garments conditioned with samples **3L** and **7LC**. This observation can be explained as a consequence of the higher adsorption of modified HEC onto the polyester surface, which results in a more efficient displacement of adsorbed ions. These findings are in agreement with that previously observed for stain removals where polyester garments conditioned with hydrophobic and amphiphilic HECs displayed a much higher efficiency in the removal of motor oil than unmodified HEC, perhaps as a result of more significant polymer deposition. Despite possessing positively charged groups, ζ-potential values of **7LC** were comparable with that observed for sample **3L**. We speculate that cationic groups do actively participate in the adsorption of amphiphilic HEC, being electrostatically attracted by the weak anionic surface of the polyester fabrics.^27^ Therefore, potentially no cationic appendage is directly exposed to the streaming medium since this would have caused a much higher increase in the observed ζ-potential. Lastly, the ζ-potential of polyester fabrics conditioned with a typical laundry detergent was found to be approximately −52 mV. The slight increase observed was probably due to moderate deposition of the nonionic surfactant onto the synthetic garments.

## Conclusions

This work confirmed that hydroxyethyl cellulose (HEC) ethers could be used as soil-release and anti-redeposition additives in a laundry formulation. The introduction of lauryl hydrophobic appendages onto HEC backbone was shown to be crucial in delivering anti-redeposition benefits (measured as textile whiteness degree) on both synthetic and cotton-based fabrics. Interestingly, fabric cleanability was strongly influenced by the degree of substitution of lauryl groups, and the highest whiteness degree was observed only within a narrow content of lauryl moieties. By contrast, the addition of cationic modifications (in the form of trimethyl ammonium chloride) resulted in poor fabric cleanability. A combined use of colorimetric and turbidity analysis allowed some light to be shed on this aspect. Turbidity studies suggested that HECs displaying cationic groups (amphiphilic HEC) are unable to maintain clay particles in suspension in the presence of surfactants. This observation, further confirmed by colorimetric analysis, can be explained as a consequence of coacervate formation between amphiphilic HEC and anionic surfactants, which leads to macroscopic phase separation. The aggregation of HECs and anionic surfactants through electrostatic interactions resulted in a lack of active specimens to maintain clay particles suspended and hence clay particles aggregated and precipitated in the washing liquor.

Interestingly, it was found that the soil-release ability of HEC hydrophobic ethers on synthetic garments was not affected by the presence of cationic moieties. Indeed, regardless of the composition of their hydrophobic\cationic appendages, hydrophobic and amphiphilic HECs displayed similar soil-release indexes, suggesting that positively charged groups do not alter the ability of hydrophobic HEC to remove the adsorbed soil. The streaming potential analysis of the surface charge of polyester fabrics conditioned with modified HECs revealed that hydrophobic and amphiphilic HECs provided polyester fabrics with similar characteristics as they both show comparable ζ-potential values. This observation suggests that all HECs, irrespective of the composition of their substituents, are able to impart polyester fabrics with similar oil-repellent finishes as a result of similar levels of deposition. Supplementary investigations are needed to further understand the deposition process of HEC ethers onto fabrics.

Overall, our results highlight a clear relation between modified HEC composition and their applicability as soil-release agent. The length of hydrophobic appendages and their degree of substitution are key factors that need to be finely tuned to deliver significant benefits on textiles. Cationic moieties should be avoided in the design of new soil-release agents as these were found to compromise the modified HEC anti-redeposition performance with particulate soil.

## Supporting Information Available

^1^H NMR spectra (700 MHz, D_2_O) of hydrophobic and amphiphilic HEC (PDF)
