# Supplementary material for: Evaluating the Role of Hydrophobic and Cationic Appendages on the Laundry Performance of Modified Hydroxyethyl Celluloses
Source: Ind Eng Chem Res. 2022 Sep 14;61(38):14159–72. doi: 10.1021/acs.iecr.2c01698 (PMC9523713; doi:10.1021/acs.iecr.2c01698)
Supplement: Supplementary file 1 — ie2c01698_si_001.pdf [file ie2c01698_si_001.pdf]

**Supporting Information**

# Evaluating the role of hydrophobic and cationic appendages upon the laundry performance of modified hydroxyethyl celluloses

**Marcellino D'Avino[a], Ruth Chilton[b], Si Gang[b], Mark R. Sivik[c] and David A. Fulton[a]***

[a] Chemistry-School of Natural and Environmental Sciences, Newcastle University, Newcastle upon Tyne, NE1 8QB, United Kingdom

[b] The Procter & Gamble Company, Newcastle Innovation Centre, Newcastle upon Tyne, NE12 9TS, United Kingdom

[c] The Procter & Gamble Company, Fabric & Home Care Innovation Centre, Cincinnati, Ohio, 45202, United States

*Email david.fulton@ncl.ac.uk

Table of contents

**Figure S1:** $^{1}$H NMR spectra of samples **3-6L**; **7-9LC**

**Figure S2:** $^{1}$H NMR spectra of samples **1E-2E**; **3EC-4EC**; **1H**; **1HC**

**Figure S3:** $^{1}$H NMR spectra of samples **1L-3L**

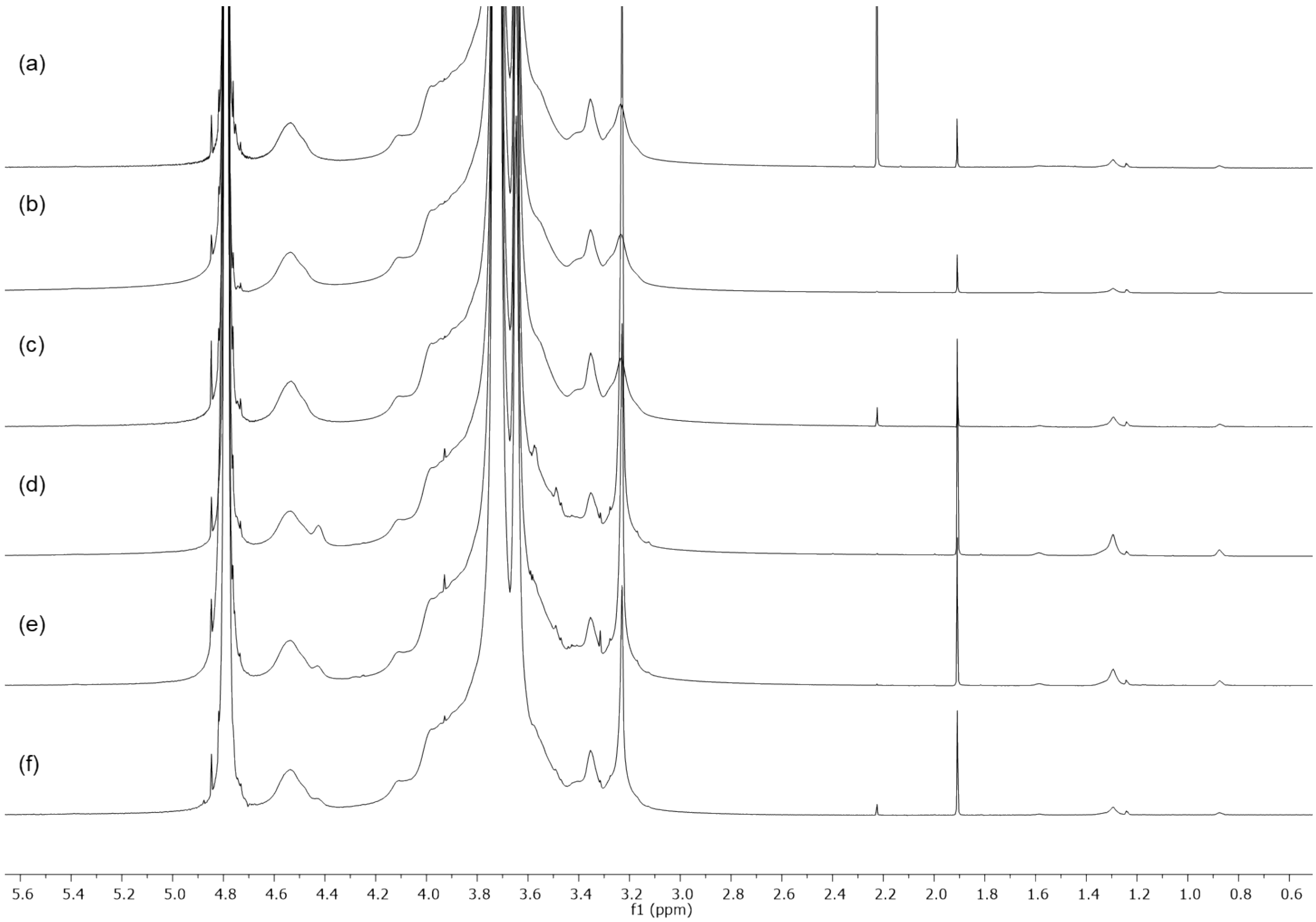


**Figure S1:** $^{1}$H NMR spectra (700 MHz, $D_2O$) of (a) sample 4L, (b) sample 5L, (c) sample 6L, (d) sample 7LC, (e) sample 8LC and (f) sample 9LC.

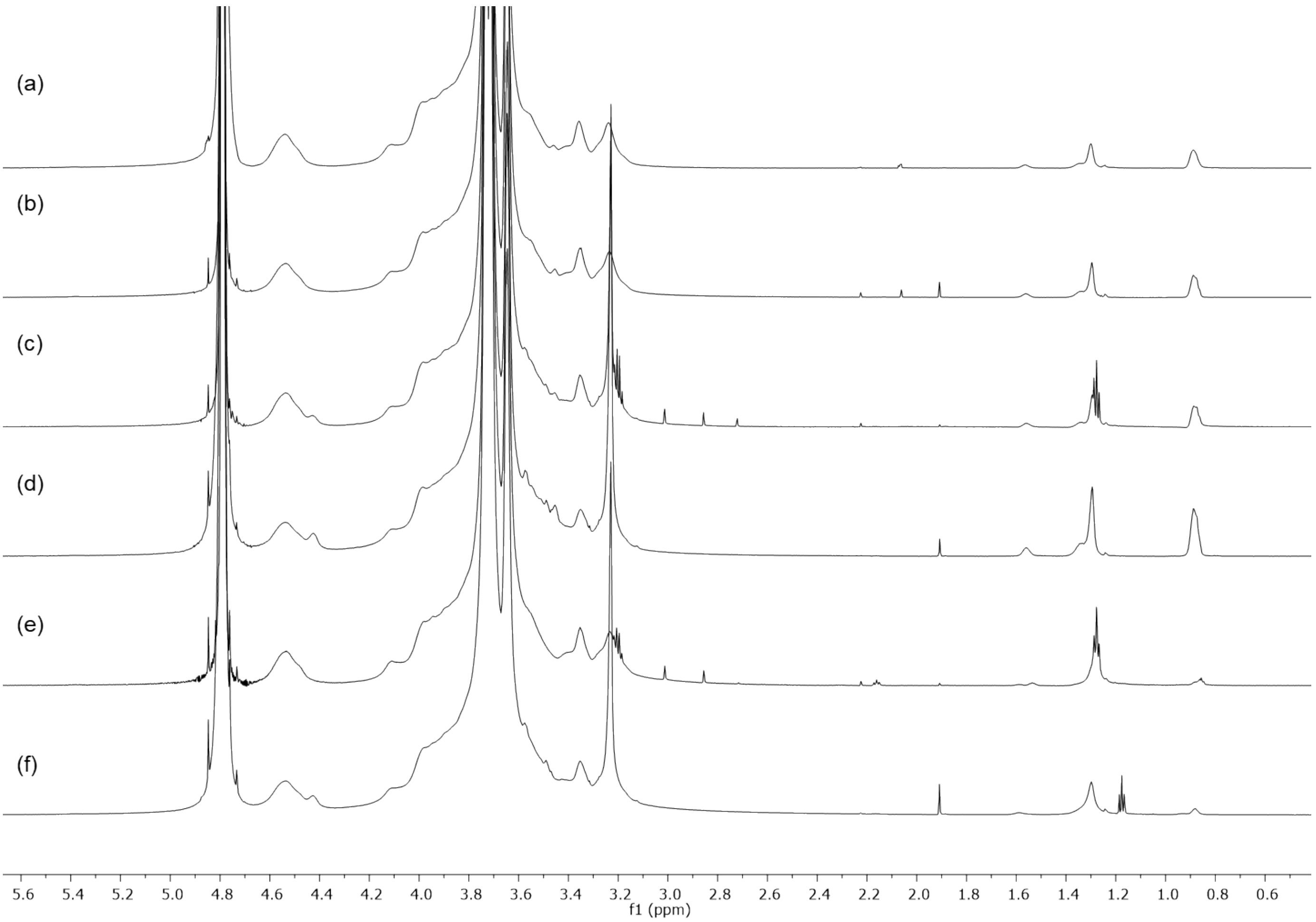


**Figure S2:** $^1$H NMR spectra (700 MHz, $D_2O$) of (a) sample 1E, (b) sample 2E, (c) sample 3EC, (d) sample 4EC, (e) sample 1H and (f) sample 2H.

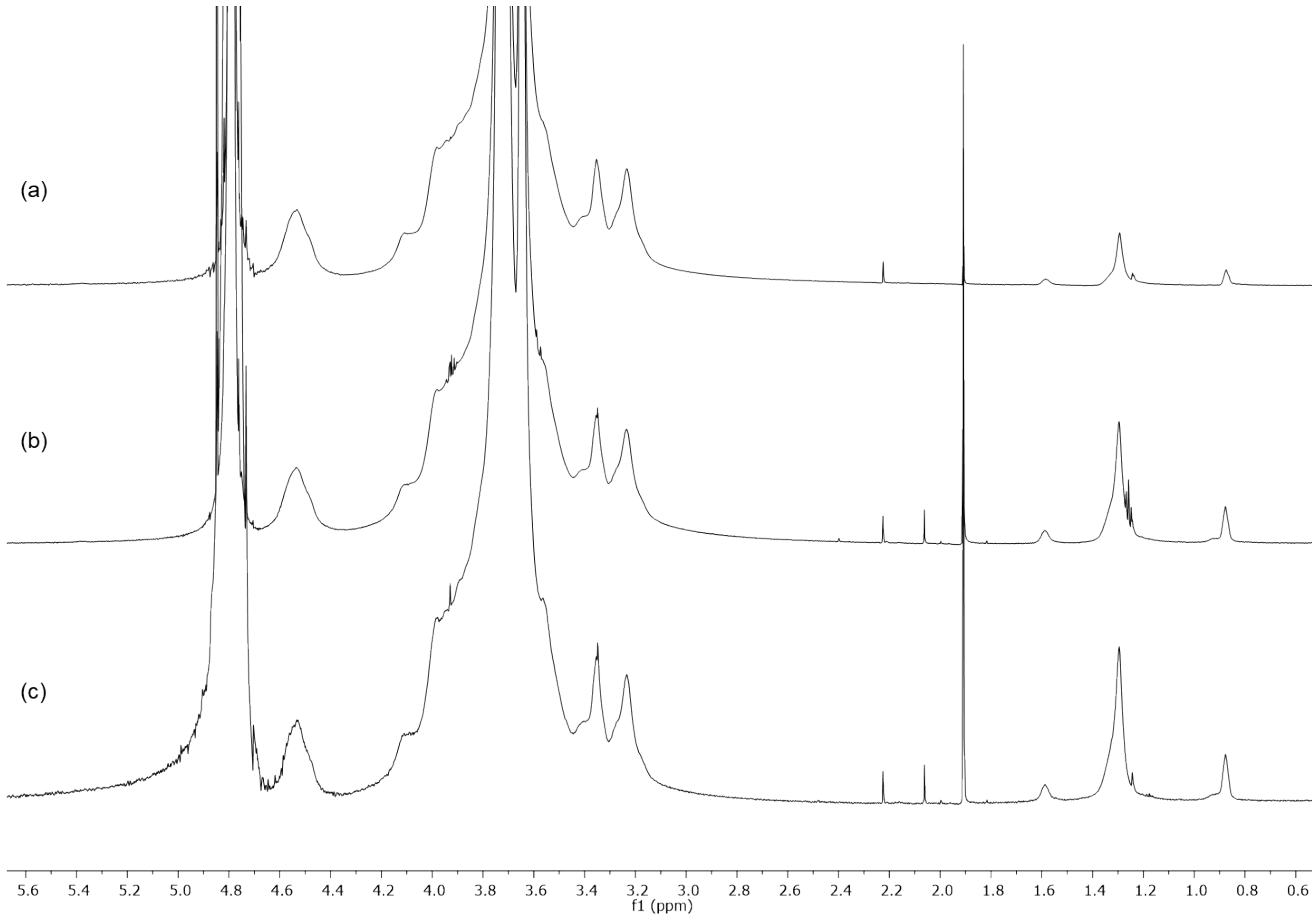


**Figure S3:** $^{1}$H NMR spectra (700 MHz, $D_2O$) of (a) sample 3L, (b) sample 2L and (c) sample 3L.
